# Geographical distribution and profile of medical doctors in public sector hospitals of the Limpopo Province, South Africa

**DOI:** 10.4102/phcfm.v9i1.1443

**Published:** 2017-09-27

**Authors:** Samuel T. Ntuli, Edwin Maboya

**Affiliations:** 1 Department of Public Health, University of Limpopo, South Africa; 2 Department of Anesthesiology, University of Limpopo, South Africa

## Abstract

**Background:**

The shortage and unequal distribution of medical doctors in low- and middle-income countries continues to be a public health concern.

**Objective:**

To establish the geographical distribution and demographic profile of medical doctors in public sector hospitals of the Limpopo Province, South Africa.

**Method:**

The PERSAL system was used to obtain information on the number of medical doctors employed in public sector hospitals of the Limpopo Province. Data were exported from PERSAL’s database and then analysed using STATA version 9.0.

**Result:**

The mean age of the 887 medical doctors was 40.1 ± 11.2 years (range 24–79 years). Sixty per cent of the doctors were male, 66% were aged ≤ 45 years and 84% were African. Most of the doctors (86%) were medical officers, of which 55% had < 5 years working experience. Overall, the doctor-to-population ratio for the five districts in the province was 16.4/100 000, with Capricorn (33.7/100 000) and Waterberg (20.2/100 000) recording the highest ratios. A large proportion (43%) of medical officers are employed in the Capricorn District, of which 71% were practising at the tertiary hospital.

**Conclusion:**

This study demonstrated a shortage and maldistribution of medical doctors in the public sector hospitals of the Limpopo Province. This has a potentially negative effect on the delivery of an appropriate and efficient healthcare service to the population and requires urgent attention.

## Introduction

The proposed National Health Insurance (NHI) by the government of South Africa (SA) is recognised as one of the best and the most responsive ways to improve the life expectancy of a population.^1^ However, this proposed free access and low cost health service in SA is now threatened by lack of infrastructure and shortage of human resources, particularly medical doctors. The shortage and maldistribution of these professionals is a serious problem worldwide,^2,3,4,5,6,7^ whereas the brain drain^8^ and ageing of the workforce are additional problems exacerbating the situation.^9,10^ In SA, the shortage and unequal distribution of medical doctors, with too few doctors servicing rural communities, is not a new phenomenon^11,12^ and has been highlighted in the national media.^13,14,15,16,17^

Several interventions such as training South Africans as doctors in Cuba, compulsory community service for graduate medical doctors, extension of internship to 2 years and the introduction of various financial incentives – such as scarce skills, rural allowances and the recruitment of Cuban doctors – have been tried in order to address shortage of doctors in remote and rural areas.^18,19,20,21^ In 2007, the SA government introduced the occupation-specific dispensation (OSD) policy, a financial incentive strategy aimed at attracting and retaining health professionals in the public health sector.

To date, it is not known whether the above initiatives are improving or worsening the availability of medical doctors in rural areas of SA, especially in the Limpopo Province. Therefore, the purpose of this study was to establish the geographical distribution and demographic profile of medical doctors in the public sector hospitals of the Limpopo Province, SA.

## Methodology

### Study design and population

The study included all medical officers (MO) (generalists) and specialists working in Limpopo’s public sector hospitals. From the PERSAL records as at January 2015, there were 766 MOs and 99 medical specialists and 22 specialist trainees in Limpopo. Medical specialists and MOs doing their 1-year community service and interns were excluded because they are undertaking training and often placed in public hospitals not of their choice.^22,23^

### Study area and setting

The Limpopo Province is predominantly rural and has an estimated population of 5.5 million people.^24^ The province is strategically located in the northern part of SA, with the Zimbabwean border to the north, Botswana to the west and Mozambique to the east. The province has five districts, namely Capricorn, Vhembe, Mopani, Sekhukhune and Waterberg. Each district is further subdivided into administrative functional municipalities. The total number of public hospitals per district is shown in Table 1. The province has one tertiary hospital, which is a complex comprising two hospitals situated 30 km apart in Polokwane and Mankweng. The complex delivers tertiary care and deals with some secondary and primary care cases.

**TABLE 1:** Number of hospitals per district.

Hospital types	Capricorn	Vhembe	Waterburg	Mopani	Sekhukhune
Tertiary	1	-	-	-	-
Regional	-	1	1	1	1
District	6	6	7	6	6

### Data collection and analysis

The PERSAL database – a personnel and salary system in the public service – was used to establish the location of the employed medical doctors. The database is the only and the most readily available source of information on health workers for use as a workforce policymaking tool, and it continues to be used to obtain estimates of the distribution of the workforce. The doctor-to-population ratio was used to gauge doctor availability relative to the population size, with low ratios being indicative of doctor need. From the South African Population Census of 2011, we obtained data on the population of each district.^24^ Personnel data were exported from the PERSAL database to a Microsoft Excel spreadsheet for cleanup and analysis.

### Ethical consideration

A descriptive study was conducted in the public sector hospitals of the Limpopo Province, SA. Ethical approval for the study was obtained from the University of Limpopo Ethics Committee – Polokwane campus (Ref: PMREC 87/2015), and permission to conduct the study was acquired from the Limpopo Provincial Research Committee (Ref: LP-2015RP9-492). To assure anonymity and confidentiality of the participants, data were analysed as a group.

## Results

**FIGURE 1:**
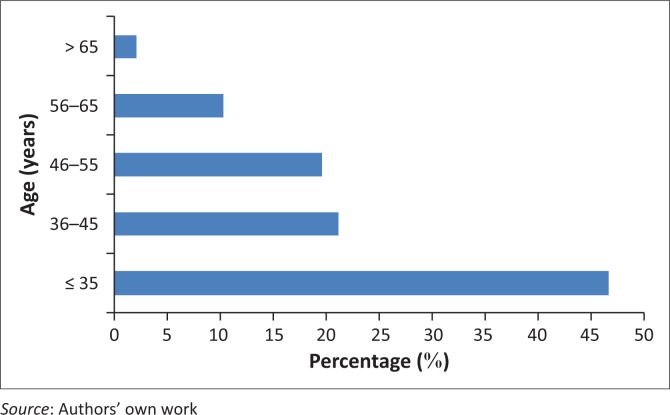
Age distribution of medical practitioners.

A total of 887 medical doctors were identified in the PERSAL system as at January 2015. Figure 1 shows the age distribution of the doctors practising in the public sector hospitals of the Limpopo Province during the study period. The mean age of these doctors was 40.1 ± 11.2 years (range 24–79 years). The majority of the doctors (66%, 599/887) were younger than 46 years. Sixty per cent of the doctors (533/887) were male and 84% (747/887) were African.

**FIGURE 2:**
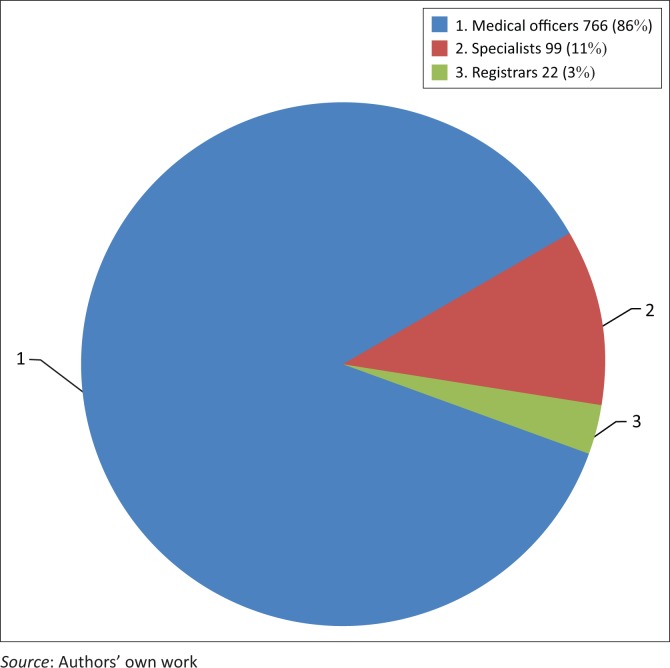
Category of doctors working in the public sector hospitals.

An analysis of each category of medical doctors is presented in Figure 2 and shows that 86% (766/887) of the doctors were MOs. More than half (55%; 421/766) of the MOs had < 5 years working experience (Grade I), 11% (81/766) had 5–10 years working experience (Grade II) and 22% (165/766) had > 10 years (Grade III) working experience. The remaining 13% (99/766) were clinical managers, of which 56% (56/99) had < 5 years working experience (Grade 1).

Table 2 shows the doctor-to-population ratio per district in the province. The doctor-to-population ratio for the province as a whole was 16.4/100 000. When the data were segregated by geographic location, the ratios, in descending order, were 33.7/100 000 for Capricorn, 20.2/100 000 for Waterberg, 9.8/100 000 for Sekhukhune, 9.7/100 000 for Mopani and 8.7/100 000 for Vhembe district.

**TABLE 2:** Doctor-population ratio in the Limpopo Province.

Districts	Population size	Number of doctors			Density per 100 000		
		All doctors^a^	Medical officer	Specialist	All doctors	Medical officer	Specialist
Capricorn	1 261 463	425	327	78	33.7	25.9	6.2
Waterberg	679 336	137	129	8	20.2	19.0	1.2
Sekhukhune	1 077 182	106	104	2	9.8	9.7	0.2
Mopani	1 092 513	106	98	6	9.7	9.0	0.5
Vhembe	1 294 796	113	108	5	8.7	8.3	0.4
Provincial	5 405 290	887	766	99	16.4	14.2	1.8

*Source*: Population size – Statistics South Africa.^24^

a All doctors includes specialist trainees.

Specialist trainees (*n* = 22): 5 in Mopani and 17 in Capricorn District.

**FIGURE 3:**
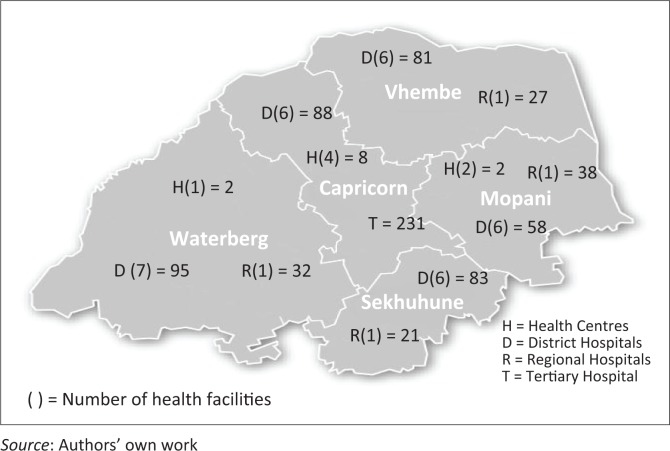
Number of medical officers practising at public hospitals per health district.

Of the 766 MOs employed within the province, 43% (327/766) were employed in the Capricorn District, of which 71% (231/327) practise within the tertiary hospital complex. In Figure 3, details about the number of MOs practising in the public hospitals of the Limpopo Province is displayed. With regard to specialists, 78% (77/99) practise within the tertiary hospital complex and 15% (15/99) practise at regional hospitals. Of the remaining specialists, some were stationed in a district hospital (2%; 2/99) and some were part of a district clinical specialist team.

## Discussion

This study explores the geographical distribution of medical doctors employed in the public sector hospitals of the Limpopo Province, SA. Recruitment and retention of a rural health workforce, particularly medical doctors, continues to be a national challenge^11,12^ and a major constraint to the delivery of essential health services.^25^ Despite comprehensive rural health workforce recruitment and retention strategies,^18,19^ rural communities continue to face a greater health workforce shortage than do their urban counterparts. In 2008, Lehmann^26^ reported an increase in the doctor-to-population ratio in the public sector hospitals of the Limpopo Province from 6/100 000 in 1994 to 17.4/100 000 in 2007. In 2010, the ratio deteriorated slightly to 17.2/100 000.^27^ Our study found that the ratio was 16.4/100 000, representing a drop of 4.7% from 17.2/100 000 in 2010.^27^ Prior to OSD policy, the Limpopo doctors were appointed on a higher notch than the doctors in other parts of SA. This could play a role in the decrease in doctor-to-population ratio and substantially reduce the shift of doctors from public to the private sector.^28^

The World Health Organization (WHO) recommends one doctor for every 1000 people. SA is below this minimum level; however, the country is much better placed than most African countries,^12,29^ but undersupplied when compared with middle-income and high-income countries.^5,12^ The national doctor-to-population ratios hide internal disparities,^30^ particularly between provinces. Evidently, in 2007, the doctor-to-population ratio in the Limpopo Province was far lower than in Western Cape and Gauteng provinces,^26,30^ which makes it difficult for Limpopo to achieve certain health intervention goals. In this study, further analysis by geographic region revealed that Capricorn and Waterberg districts have the largest doctor-to-population ratio when compared to the other three districts in the province. This might be related to regional social and economic development taking place in Capricorn^31^ and the proximity of the Waterberg District to Gauteng – which is the economic hub of SA.^32^

In many countries maldistribution is arguably a more pressing problem than absolute scarcity.^33^ For example, in Nigeria,^34^ Ghana^35^ and Peru,^36^ doctors are more likely to favour a job in urban areas over rural settings. SA is not an exception; rural doctor shortage has been documented for decades.^11^ Several interventions have been implemented to address shortages of these health professionals in remote and rural areas, such as compulsory community service for graduate medical doctors.^18,19^ This approach has had a short-term impact on the shortage of doctors in underserved communities, because many doctors leave the rural areas after completing their obligation time,^37^ whereas those who decide to stay lack the knowledge and skills in the management of obstetric and medical emergencies, as well as basic anaesthetic skills.^22^

Furthermore, the government introduced various financial incentives for health workers practising in remote and rural areas. However, financial incentives are not the only factor influencing career choice^18,19,25,38,39^ and their design and implementation is also a challenge. In this study, it was found that more than two-thirds (78%) of the specialists and almost half (43%) of MOs were clustered in the Capricorn District, of which 73% of the MOs were working in a tertiary hospital complex. This large number of MOs working in the tertiary hospital complex might be related to availability of resources, academic activities and immediate access to consultants.

Evidence from local and international studies has shown that medical school initiatives to train rural medical practitioners have successfully addressed the shortages of medical doctors in remote and rural communities.^33,40,41,42,43,44,45^ Plans for the establishment of a new medical school at the University of Limpopo might help to ease the burden of a shortage of medical doctors in this rural province. However, there are well-documented barriers to expanding the rural health workforce supply which include lack of management capacity, job satisfaction, lack of suppliers or equipment and poor working conditions.^28,29,30,39,46,47,48,49^ Unless these issues are addressed, no matter how many graduates with a rural background are produced, the problem of retaining health professionals in rural hospitals will continue. Moreover, the implementation of OSD policy appears to have reduced the migration of healthcare workers because of low salaries.^48,50,51^ However, this study showed that the maldistribution of healthcare workers remains a major problem in underdeveloped districts. The ‘Deprived Area Incentive Scheme’ in Ghana and the ‘Health Workers Rural Retention Scheme’ in Zambia, which includes housing or housing allowances, fast-track promotion and career development opportunities, car or car loans and education grants for staff children among other things, are successful strategies used in keeping doctors in underserved areas.^52^ Thus, these schemes can be tested in this rural province.

### Study limitations

The study used only doctor-to-population ratio for assessing maldistribution of doctors. The ratio was not adjusted by healthcare needs, health status and health service utilisation. In addition, the findings of this study cannot be considered representative of all the provinces in SA. The study was based on the PERSAL data only and was not verified with the institutions directly. It is assumed that a number of specialist trainees (registrars) were still in MOs category in the PERSAL system. Furthermore and more importantly, a significant number of private providers are contracted to work within the public sector on a session basis. The data could not accurately separate the number of private doctors engaged in public sector activity.

## Conclusion

This study demonstrated that there is substantial inequality in the distribution of medical doctors in the public sector hospitals of the Limpopo Province. It was demonstrated that the distribution of medical doctors is clustered in Capricorn and Waterberg Districts. It is also clear that a greater proportion of generalists are concentrated within the tertiary hospital complex, which is disadvantageous for the quality and availability of healthcare in the rural areas of the province. There is no single solution to this multifactorial problem; however, efficient use of the existing workforce and addressing the non-financial issues can be adopted as a short-term response to this challenge, whereas introduction of the schemes mentioned above should be seen as long-term strategies.
